# A qualitative study of refugee families’ experiences of the escape and travel from Syria to Sweden

**DOI:** 10.1186/s13104-018-3702-1

**Published:** 2018-08-17

**Authors:** Elisabeth Mangrio, Slobodan Zdravkovic, Elisabeth Carlson

**Affiliations:** 1 Department of Care Science, Faculty of Health and Society, Malmö University, Malmö, Sweden; 2 Malmö Institute for Studies of Migration, Diversity and Welfare (MIM), Malmö University, Malmö, Sweden

**Keywords:** Armed conflict, Escape, Migration, Public health, Refugee, Syria

## Abstract

**Objective:**

Research shows that, depending on the route of travel during the escape, the journey presents the refugees with different health risks. Traumatic events during flight may have long-lasting physical and psychological effects on the refugee children. Therefore, it is important to illuminate the experiences that refugee families arriving in Sweden have endured during their flight. A qualitative study was conducted through interviews with fifteen recently arrived Syrian refugee families.

**Results:**

The parents described different reasons as to why they as families had to escape the war. Some families had lost jobs and loved ones in the war and did not want their children to die as well. They mentioned that the journeys varied between 10 and 40 days and were usually filled with struggles and threats. The escape to Sweden was expressed as an emotionally trying journey. Many parents talked about the fear and terror the children felt. Traumatic events during the escape, such as separation from family, death of family members, sexual violence, kidnapping or extortion may have long-lasting physical and psychological effects on the refugee children and their families. Therefore, health care workers meeting and caring for these families after arrival must pay close attention to that.

## Introduction

Transnational migration occurs for diverse reasons and usually entails crossing of cultural as well as political boundaries, which requires a range of accommodation, and engaging with complex networks of power, different social and cultural systems and diverse populations [1]. The process of displacement will inevitably lead to an added health and social burden for the recipient country. In general, an individual will flee when the integrity of their person is threatened towards countries where they expect the conditions to be better. However, for many displaced refugees, movement merely means a shift from one poor and vulnerable situation into other vulnerable situations and circumstances [2].

The actual process of moving and displacement can lead to health-related difficulties, particularly for vulnerable groups such as children, women and elderly, in particular lack of vital survival necessities such as food, shelter and water required to sustain basic health. To add to this, there could be a lack of access to emergency health care in times of need as well as poor sanitary conditions that refugees have to endure during the displacement and flight [2].

Furthermore, depending on the route of travel during the flight; the journey presents the migrants with different health risks [3]. During the crossing of the sea between Turkey and Greece, many children have drowned when overcrowded boats have capsized. Infants that are born during the journey are at an increased risk of hypothermia, septicaemia, meningitis and pneumonia. Infants can also suffer from poor nutrition and diarrheal diseases and skin infections due to overcrowded accommodation and substandard hygiene. Traumatic events such as separation from family, death of family members, sexual violence, kidnapping or extortion may have long-lasting physical and psychological effects on the refugee children including depression and post-traumatic stress disorder (PTSD) [3].

The study by Hopkins and Hill describes the experiences of unaccompanied refugee children during the migration process to the UK [1]. They explained the journey with details such as trauma, fear and uncertainty brought on by the complex and traumatic circumstances during the flight. The experiences told a story of being accompanied by mostly male agents. The agents were not seen as protectors but as abusers, and some of the agents even let, their friends abuse the children. Rape was often a part of the price of being brought to the recipient country [1].

The Syrian War started through anti-government demonstrations in 2011 and escalated through opposition groups fighting back [4]. More than 11 million have been killed or forced to flee their homes, including 5.3 million people taking refuge in neighbouring countries. 3.3 million Syrians have fled to Turkey and some of these have chosen to continue to Greece and Europe through crossing the Mediterranean Sea [4]. During 2015, approximately 270,000 refugees crossed the Mediterranean Sea and around 2700 died along the way [5]. When refugees reach Sweden and until they have obtained refugee status, they are entitled necessary medical care [6]. During the last years, between 2015 until now, Sweden has received around 220,000 refugees [7]. Based on earlier research indicating the exposed and vulnerable situation experienced by refugees during transition, in particular children and women, this current study focuses on the experiences of families with children fleeing from Syria to Sweden.

## Main text

### Method

This qualitative descriptive study was conducted through interviews with 15 recently arrived refugee families that had received their residence permit and how they experienced their situation during the resettlement process. This Research Note focuses on the experiences these families endured en route to Sweden.

#### Participants

The inclusion criteria consisted of recently arrived refugee families who had applied for asylum and received their residence permits. In Sweden, this implies that these families had obtained refugee status and started the establishment process. The researchers worked together with civic and health communicators within the local community from the provincial government which formed a link between recently arrived refugee families and workers within health and social care in the society [8]. Through these communicators, eligible families were asked about participation for interviews. The parents in the families were between 21 and 65 years of age and had 1–6 children per family. They had been in Sweden between 2 and 36 months.

#### Data collection

The interviews were performed, when possible, with both parents present. No children participated in the interviews. The interviews started with an open question asking if the family could describe the escape to Sweden. Furthermore, the interview guide covered topics such as how the family perceived their current situation, health, struggles and hope for the future. The interviews were recorded and transcribed verbatim, shortly after each interview. The interviews averaged 36 min (minimum 17 and maximum 60 min).

#### Data analysis

The transcribed interviews were analysed with content analysis by Burnard [9]. Open coding was performed in English and was used to create categories and three categories were formed. To ensure credibility, triangulation, a method for cross-checking data from the perspective of multiple researchers was performed, and emerging codes and categories were discussed until we reached consensus [10]. Extracts from the interviews, explaining the analysis from meaning unit to categories are illustrated in Table 1.

**Table 1:** Extracts from the interviews, explaining the analysis from meaning unit to category

Meaning unit	Code	Category
We had no work since we lost our jobs and then we realised that we would have to escape from the country (3)	No work and had to escape	The reason behind the escape
It was a tough and difficult route and the weather conditions were bad with rain and wet ground to walk on (6)	Tough route with rain and wet ground	The escape through Europe
A really difficult trip and by a miracle we escaped death (11)	Difficult trip and by a miracle we escaped death	The emotional experience of the escape

### Results

After analysis the following three categories were formed: *The reason behind the escape; The escape through Europe; The emotional experience of the escape.*

#### The reason behind the escape

The parents described different reasons behind why they as families had to escape the war-zone in Syria. They described their situation in Syria and the difficulties brought on by the war. One family mentioned that they lost their jobs due to the war and therefore had to escape. Another family mentioned that family members had already died in the war and that they now wanted to escape and avoid the same happening to their children:

“*I have already lost two of my brothers in the war and these brothers had their own children. I thought that I did not want to lose my children because of the war, so we escaped. The situation in the country got worse and the children could not attend school any longer”.* Informant 6.

Some families had first escaped to Lebanon in order to avoid the war and told about the difficult circumstances they had to suffer due to severe living difficulties and how the children were just allowed to attend school in the evenings. They were treated harshly by the Lebanese citizens and were forced to give away their money. One parent explained how a curfew after 6 p.m. was imposed on Syrian families, and how the police randomly showed up at the door, breaking it and forcing themselves into the house threatening the families to leave Lebanon. This was put forward as a reason why people left as soon as the migrant route through Europe opened.

#### The escape through Europe

A few families talked about travelling to Sweden legally by obtaining a visa, but most families arrived via illegal means and had to be smuggled into Sweden having crossed countries such as Turkey, Greece, Macedonia, Serbia, Croatia, Hungary, Austria and Germany. The duration of these journeys varied between 10 and 40 days and they were usually filled with struggles and threats. The families had to walk long distances on foot through rain and bad weather conditions. One family told about the health issues such as diabetes and asthma that were worsened during the escape due to travel and stress along the way. A majority of the families mentioned the sea crossing between Turkey and Greece and the struggle and deaths that it brought and they called it the death trip:

“*During the trip over the sea, I lost the rest of my family, since the boat got full of water and we had to find our way to escape onto an island. I was there together with other people on the island and we were finally found by the Italian coast guards, but a lot of people died during this trip”,* Informant 9.

A mother told about how she was travelling with a few of her children crossing the sea and spoke of the feelings such a journey evokes, in particular the fear experienced during night-time. Other situations of difficulties were expressed such as being held captive by the police along the route. Even though most of the interviewed families escaped together as a family, some of them had sent their children ahead of them and were later united in Sweden.

#### The emotional experience of the escape

The escape to Sweden was expressed as an emotionally trying journey. Many parents talked about the fear and terror their children felt along the way. One father told about how the police in Serbia beat him and what his children must have felt witnessing the abuse. Other families talked about the harsh and tough escape and explained how it felt like a miracle that they were now alive. One father expressed the feelings about the trip as follows:

“*To talk about this trip is easy, but to experience it is so much more difficult”,* Informant 3.

Many of the interviewed families mentioned that they were still mentally affected by memories that the escape provoked in their minds. Another father expressed feelings of fear and threats along the way and how in Serbia they were haunted by gangs. Many only felt relief after entering into Germany, and only then felt that they would finally be fine.

### Discussion

The results showed that the escape and the travel through Europe were filled with danger, threats, and risk of death when crossing the sea between Turkey and Greece. It is already known that the flight route presents the migrants with health risks [3]. In this case, it could be health risks related to the long distance that the families had to travel as well as physical fatigue since they had to walk for long distances. The travel also involved mental risks since the families experienced threats and persecution when crossing the borders and saw people losing their lives along the way, as well as losing family members during their flight. The International Society for Social Paediatrics and Child Health (ISSOP) migration working group [3] states that traumatic events such as separation from family, death of family members, sexual violence, kidnapping or extortion may have long-lasting physical and psychological effects on the migrant children, including depression and PTSD [3]. This needs to be considered by health care workers meeting and caring for these families after arrival, since the possible life-threatening events during the escape probably affect the physical and mental health status of the family for a substantial amount of time. Doctors and nurses within primary health care settings need to observe symptoms of depression and PTSD in adults as well as in children and should create an awareness about the health-effects that the escape and travel could have [3]. Thomas and Thomas [2] encourage recipient countries to practice health-assessment after arrival as well as health promotion and health education after arrival, in order to address health issues after escape. Health screening is a part of the introduction programme for refugees in Sweden although not everyone attends it [11]. Therefore, the society should prioritize initiatives directed towards increasing the attendance of refugees including adequate health information to both refugee children and adults in order for them to understand the importance of health services as well as maintaining health [3].

### Conclusions

It is of great importance to pay attention to the refugee families’ experiences of the escape and travel through Europe. Since life-threatening events along the escape route can affect the mental and physical health of these families for a long time after arrival in Sweden, attention needs to be directed towards this group in society. For this reason, health care professionals working at primary health care centres, and school nurses in particular, need to pay attention to the health situation of refugee children and their families as they are in a position where they can build trusting relationships with these families.

## Limitations

The interviews were conducted mostly in Swedish with Arabic interpreting, apart from two interviews that were conducted in Swedish and English. The interpreters were all authorised. Interpreting increases a researcher’s potential study population but poses challenges in terms of quality [12]. According to Squires [13], the credentials of an interpreter affect the qualitative data and analytical process and it is therefore recommended to use an authorised translator. Interviews that are interpreted may be affected by different coherence systems, meta-communication and the subtle ways that participants and interpreters perceive each other [12]. All this needed to be considered when analysing the results of this study and optimising the outcome and reliability of the interviews [12]. Since the researchers are aware of the war-situation in Syria and exposed to continuous media reports we were aware of the need for reflexivity during the research process. Reflexivity has been described as conscious self-awareness of subjective responses and inter-subjective dynamics. Following interviews and during analysis the research team discussed the experiences made and the emerging findings, this process ensured that methodological rigour was developed, which is necessary for trustworthy qualitative results [14]. Although the aim of the study was to focus on the families during the route of travel, only the parents’ experiences were derived from the interviews. However, earlier research shows that the mental and psychosocial health of refugee children depends on the mental health of their parents during the migration process [3]. It could be beneficial to interview the children in order to get more of their perceptions and experiences from the route of travel.

## Abbreviations

EM: Elisabeth Mangrio
EC: Elisabeth Carlson
SZ: Slobodan Zdravkovic
AMIF: Asylum, Migration and Integration Fund
ISSOP: International Society for Social Paediatrics and Child Health
PTSD: post-traumatic stress disorder
